# Heterogeneity in medical coping modes and the moderating role of social support on social disability in patients after percutaneous coronary intervention

**DOI:** 10.3389/fpsyg.2025.1731898

**Published:** 2025-12-17

**Authors:** Jia Zhang, Song bin Huang, Dan ni Peng, Ling ling Yang, Fei wan Huang, Yan jie Chen

**Affiliations:** Heyuan People's Hospital, Heyuan, China

**Keywords:** latent profile analysis, medical coping modes, moderation analysis, percutaneous coronary intervention, social disability, social support

## Abstract

**Objective:**

This study aimed to identify heterogeneous patterns of medical coping modes (MCM) and to examine the moderating role of social support in the relationship between these patterns and social disability in young and middle-aged patients after percutaneous coronary intervention (PCI).

**Methods:**

A cross-sectional study was conducted among 129 post-PCI patients from a single center in China. Participants completed the Medical Coping Modes Questionnaire (MCMQ), the Social Support Rating Scale (SSRS), and the Social Disability Screening Schedule (SDSS). Latent profile analysis (LPA) was used to identify distinct coping patterns. The moderation effect of social support was tested using the Johnson-Neyman technique.

**Results:**

Two distinct coping profiles were identified via LPA: “Adaptive Copers” (55.1%), characterized by higher confrontation and lower avoidance/resignation, and “Maladaptive Copers” (44.9%), showing the opposite pattern. A counterintuitive finding emerged, with the Maladaptive Copers reporting significantly lower social disability scores. Furthermore, beyond this profile differentiation, social support demonstrated a significant U-shaped moderating effect in the coping-disability relationship. Its moderating role was statistically significant only at very low (<39.884) and very high (>52.924) levels of support.

**Conclusion:**

This study reveals two key findings: first, post-PCI patients are heterogeneous in coping, comprising adaptive and maladaptive subgroups; second, the impact of these coping styles on social disability is non-linearly moderated by social support. Clinicians should assess both coping profiles and social support levels to tailor interventions effectively.

## 1 Introduction

Percutaneous coronary intervention (PCI) is a critical revascularization procedure for patients with acute myocardial infarction (AMI), significantly reducing mortality and improving cardiac outcomes (Levine et al., 2021). However, the physical recovery post-PCI is often accompanied by substantial psychological challenges and adjustments in social functioning (Zhang et al., 2019). The manner in which patients cope with their illness plays a pivotal role in determining their long-term psychosocial adaptation and quality of life (Feifel et al., 1987).

Medical coping modes (MCM), typically categorized into confrontation, avoidance, and resignation, represent the cognitive and behavioral efforts patients employ to manage the specific stressors related to their health condition (Carver et al., 1989). The process of medical coping places distinct cognitive and behavioral demands on patients, necessitating a level of health literacy to effectively understand medical information, communicate with providers, and make informed decisions about their care—a set of challenges not typically required in coping with ordinary social stressors (Berkman et al., 2010). Research indicates that coping strategies are not uniform across cardiac patients and can significantly influence functional recovery, including a return to social roles and activities (Kristofferzon et al., 2005; Tiemensma et al., 2016). Social disability, referring to the impairment in performing usual work, social, and family activities due to health problems, is a crucial outcome measure post-PCI (Janca et al., 1996). High levels of social disability can hinder a patient’s successful reintegration into society, thereby increasing the risk of depression and reducing overall well-being (Frasure-Smith and Lesperance, 2008). Prior studies have established a direct link between maladaptive coping strategies, such as avoidance and resignation, and poorer functional outcomes in various chronic illnesses (Richardson et al., 2012; Am et al., 2012; Mavrides and Nemeroff, 2013). In this study, we aimed to investigate the direct relationship between MCM and social disability in young and middle-aged post-PCI patients. Furthermore, previous research has suggested the existence of distinct, heterogeneous patterns of coping among patients with chronic diseases (Leventhal et al., 1980; Hagger et al., 2017; Berg et al., 2018). To explore potential subgroups, we employed latent profile analysis (LPA).

In clinical practice, the provision of adequate social support is considered essential for promoting positive recovery outcomes. Social support, defined as the perceived availability of assistance from one’s social network, can buffer the negative psychological impact of stressful events (Cohen and Wills, 1985). According to the Stress-Buffering Hypothesis (Holt-Lunstad et al., 2015), social support can mitigate the adverse effects of stress on health by providing resources (e.g., emotional comfort, tangible aid) that strengthen an individual’s capacity to cope. Grounded in this theory, we posit a moderation model where social support (the external resource) alters the strength of the relationship between coping modes (the internal process) and social disability (the outcome). While social support is generally viewed as beneficial, its protective effect may vary in strength, potentially operating differently across subgroups of patients with distinct coping profiles. A previous study also indicated that low social support was associated with greater functional impairment in cardiac patients (Burg and Abrams, 2001). Therefore, social support may moderate the effects of MCM on patients’ social disability.

However, current research on coping and social disability in PCI patients has often treated coping as a uniform construct, with limited exploration of how naturally occurring coping patterns might interact with social support. Therefore, we propose the following hypotheses:

(1) There is a substantial correlation between MCM and social disability.
(2) Several distinct patterns of MCM will be recognized in post-PCI patients.
(3) Social support moderates the relationship between the identified MCM profiles and perceived social support.

## 2 Methods

### 2.1 Design and participants

This study employed a cross-sectional design and utilized moderation analysis. A convenience sampling method was used to recruit young and middle-aged patients with acute myocardial infarction (AMI) who underwent their first percutaneous coronary intervention (PCI) at the Department of Cardiology, Heyuan People’s Hospital between August 2022 and April 2024.

The inclusion criteria were as follows:

(1) Met the diagnostic criteria for AMI as outlined in the guidelines for the rapid diagnosis and treatment of acute coronary syndrome in emergency settings (Rao et al., 2025);
(2) Had successfully undergone primary stent implantation for the first time, with cardiac function classified as New York Heart Association (NYHA) Class II or above (Yancy et al., 2013), and categorized as low to moderate risk according to the cardiac rehabilitation risk stratification;
(3) Were in a stable postoperative condition and met discharge criteria;
(4) Aged between 18 and 59 years;
(5) Voluntarily agreed to participate in the study and provided written informed consent.

The exclusion criteria were as follows:

(1) Allergy or intolerance to medications used in standard therapy;
(2) Comorbid severe diseases of other organs or systems (e.g., severe hepatic or renal insufficiency);
(3) Severe cardiac insufficiency (NYHA Class III-IV; Yancy et al., 2013) unimproved by optimized medical therapy;
(4) Presence of severe complications such as long-term steroid use, trauma, recent major surgery, systemic infection, severe anemia, or cachexia.

These specific criteria were implemented to homogenize the sample and focus the investigation on a typical working-age population experiencing their first major cardiac event, thereby allowing for a clearer examination of the relationships between coping modes, social support, and social disability in this distinct subgroup.

Initial data screening revealed that 19 questionnaires had significant missing data. After excluding these cases, a final sample of 129 participants was included in the analysis (response rate = 93.5%).

### 2.2 Measures

#### 2.2.1 Demographic and clinical characteristics

A self-designed data collection form was used to gather information on demographic and clinical variables, the selection of which was aligned with established practices in cardiovascular clinical research and guideline recommendations (Yeh et al., 2016; Lawton et al., 2022). The specifically collected variables included demographic characteristics—age, gender, education level, and type of medical insurance, as well as clinical variables such as length of hospital stay (in days) and a documented history of hypertension, hyperlipidemia, and diabetes mellitus. Demographic data were obtained through patient interviews conducted prior to discharge, while all clinical data were extracted from the electronic medical records system by the research team.

#### 2.2.2 Medical coping modes

The Medical Coping Modes Questionnaire (MCMQ) developed by Feifel et al. (1987) was used to assess patients’ coping strategies. The scale consists of three subscales: confrontation, avoidance, and resignation. It comprises 19 items rated on a 4-point Likert scale. Higher scores on each subscale indicate a greater tendency to use that specific coping style. The Chinese version of the MCMQ has demonstrated good reliability and validity (Shen and Jiang, 2000). Te Cronbach’s *α* value was 0.820.

#### 2.2.3 Perceived social support

Perceived Social Support was measured using the Social Support Rating Scale (SSRS) developed by Xiao (1994), which assesses an individual’s subjective perception of the availability and adequacy of social support. The scale includes 10 items assessing three dimensions: objective support, subjective support, and support utilization. The total score ranges from 12 to 66, with higher scores indicating higher levels of social support. The scale has been widely used in Chinese populations with good reliability (Wang et al., 2018). Te Cronbach’s *α* value was 0.911.

#### 2.2.4 Social disability screening schedule

Social Disability Screening Schedule (SDSS; Janca et al., 1996; Zhang, 1998) was used to assess functional impairment in social life. The scale contains 10 items measuring disability across three specific domains: (1) work and school performance, (2) social and leisure activities, and (3) family and home responsibilities. Each item is rated on a 3-point scale (0 = no disability, 1 = mild to moderate disability, 2 = severe disability). The total score ranges from 0 to 20, with higher scores indicating greater social disability. The Chinese version has shown good psychometric properties (Phillips et al., 2009). Te Cronbach’s α value was 0.902.

### 2.3 Data analyses

Data analysis was performed using SPSS Statistics 23.0 and the PROCESS 3.6 macro for SPSS developed by Hayes (Hayes, 2013), *p* < 0.05 was considered statistically significant for all analyses. The specific analytical steps were as follows:

(1) Descriptive statistics were computed, encompassing means, standard deviations, frequencies, and percentages for all demographic, clinical, and main study variables (MCMQ, SSRS, and SDSS scores).
(2) Univariate analyses were conducted using independent samples t-tests and one-way analysis of variance (ANOVA) to examine if social disability scores (SDSS) differed across various demographic and clinical subgroups. This step aimed to identify potential confounding factors for consideration.
(3) The normality of MCMQ, SSRS, and SDSS total scores was assessed using the Shapiro–Wilk test to inform the choice between Pearson or Spearman correlation for each bivariate relationship.
(4) Latent profile analysis (LPA) was employed to identify heterogeneous subgroups based on participants’ responses to the three subscales of the MCMQ: confrontation, avoidance, and resignation (Zhao et al., 2023; Teng et al., 2024; Journault et al., 2022). Model fit was assessed using the Akaike Information Criterion (AIC), Bayesian Information Criterion (BIC), and sample size-adjusted BIC (aBIC; Berlin et al., 2014).
(5) An independent samples t-test was used to compare social disability scores (SDSS) between the coping profiles to determine if coping patterns were associated with different levels of functional outcome (Kelter, 2020).
(6) The moderating effect of perceived social support (SSRS) on the relationship between coping profiles and social disability was tested using the Johnson-Neyman technique via the PROCESS macro (Bauer and Curran, 2005), which precisely delineates the regions of significance for the moderator.

### 2.4 Ethical considerations

This study was approved by the Ethics Committee of Heyuan People’s Hospital (Approval No. YXLL-2021 K53). The trial was also registered with the Chinese Clinical Trial Registry (Registration No. ChiCTR2500098631). All procedures performed in studies involving human participants were in accordance with the ethical standards of the institutional and/or national research committee and with the 1964 Helsinki declaration and its later amendments or comparable ethical standards. Written informed consent was obtained from all individual participants included in the study. All participants were assured of data confidentiality and anonymity.

## 3 Result

### 3.1 Demographic characteristics

A total of 129 post-PCI patients were included in this study. As shown in Table 1, the sample had a mean age of 51.2 ± 7.1 years and was predominantly male (93.0%). No significant differences in social disability scores were found across most demographic and clinical variables (all *p* > 0.05).

**Table 1:** Demographic analysis among patients after percutaneous coronary intervention.

Variables	Outcome variable: social disability		
	M ± SD	n(%)	*p* value
Age	51.2 ± 7.1		
Length of hospital stay**(d)**	6.8 ± 3.3		
Gender			0.897
Male	1.89 ± 2.59	120(93.0)	
Female	1.78 ± 1.71	9(7.0)	
Education			0.248
Junior high school or below	1.85 ± 2.54	74(57.3)	
High school (including vocational school)	2.32 ± 2.73	37(28.7)	
College degree or above	1.11 ± 1.97	18(14.0)	
Medical Insurance			0.099
No insurance	1.33 ± 2.31	3(2.3)	
Urban residents’ insurance	2.08 ± 2.64	88(68.2)	
Employee Medical insurance	1.47 ± 2.31	38(29.5)	
Hyperlipidemia			0.058
No	2.15 ± 2.74	92(71.3)	
Yes	1.22 ± 1.81	37(28.7)	
Hypertension			0.535
No	1.80 ± 2.53	96(74.4)	
Yes	2.12 ± 2.60	33(25.6)	
Diabetes Mellitus			0.479
No	1.96 ± 2.65	104(80.6)	
Yes	1.56 ± 2.00	25(19.4)	

### 3.2 Descriptive statistics for main study variables

The scores for the main study variables are presented in Table 2. Given the non-normal distribution of MCMQ and SDSS, their scores are reported as median and interquartile range (IQR): the median MCMQ score was 38.00 (IQR = 34.00–47.00), and the median SDSS score was 1.00 (IQR = 0.00–3.50). The SSRS score, which was normally distributed, is reported as mean ± standard deviation (39.76 ± 6.66).

**Table 2:** Correlation analysis of MCMQ, SSRS and SDSS.

Variable	Median (IQR) / Mean ± SD	1	2	3
1. MCMQ	38.00(34.00–47.00)	-	0.571**	−0.526**
2. SSRS	39.76 ± 6.66	0.571**	-	−0.631**
3. SDSS	1.00(0.00–3.50)	−0.526**	−0.631**	-

Data for normally distributed variables (SSRS) are presented as Mean ± Standard Deviation; data for non-normally distributed variables (MCMQ, SDSS) are presented as Median (Interquartile Range). All correlations are Spearman’s rank-order correlation coefficients (*p*). *p < 0.05, **p < 0.001.

### 3.3 Correlation analysis of main variables

As shown in Table 2, MCMQ showed a significant positive correlation with SSRS (*p* = 0.571, *p* < 0.001), and a significant negative correlation with SDSS (*p* = −0.526, *p* < 0.001). Similarly, SSRS was negatively correlated with SDSS (*p* = −0.631, *p* < 0.001).

### 3.4 Latent profile analysis of medical coping modes

**Figure 1:**
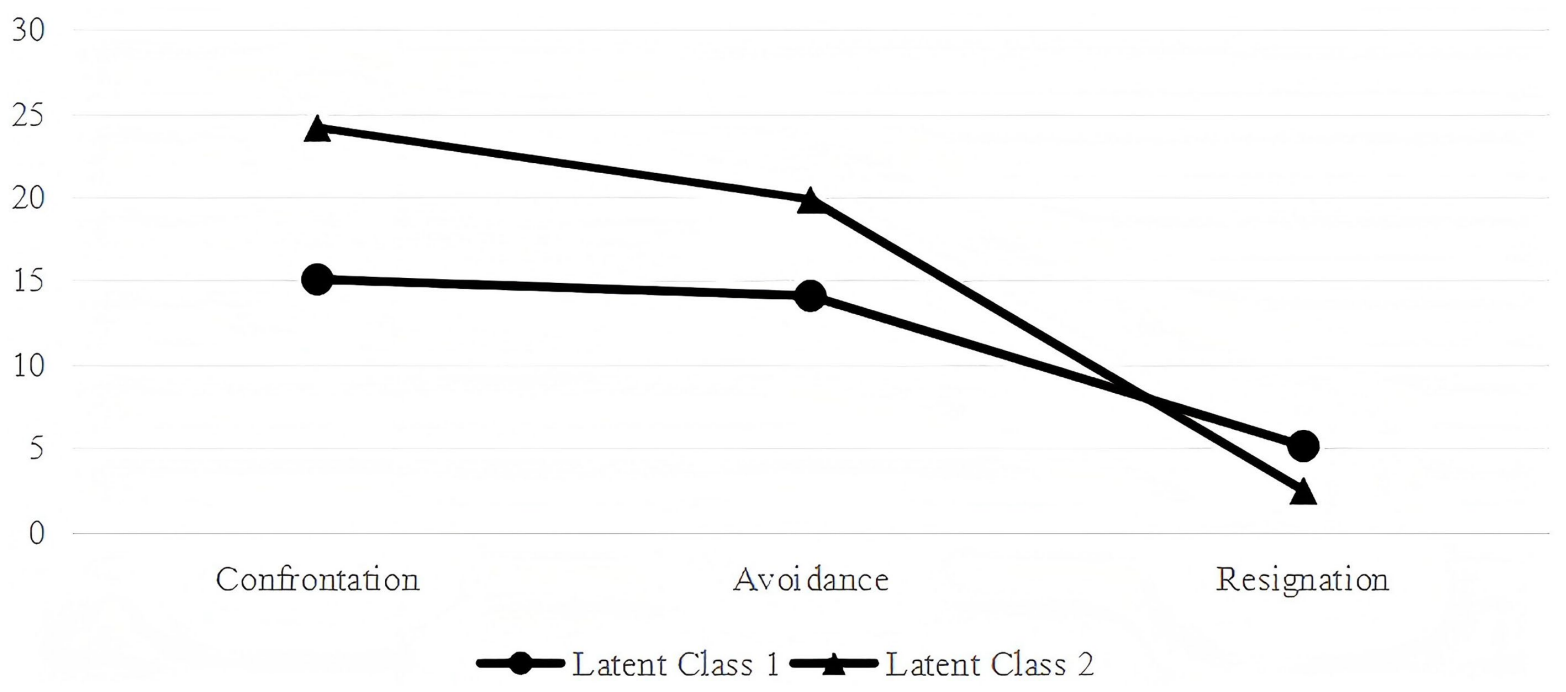
Characteristics of two latent profiles of medical coping modes in PCI patients.

Latent profile analysis (LPA) was employed to identify heterogeneous patterns in medical coping modes among PCI patients. Fit indices for the competing models are presented in Table 3. The 2-class solution was selected as the optimal model based on the lower AIC, BIC, and aBIC values compared to other models. Additionally, this model demonstrated a high entropy value, and both the LMRT (*p* < 0.001) and BLRT (*p* < 0.001) reached statistical significance. According to the characteristics of the model (Figure 1), the subgroups were named as adaptive copers (Class 1, 55.1%), and maladaptive copers (Class 2, 44.9%).

**Table 3:** Fit indices for latent profile analysis models of medical coping modes.

Latent Class	AIC	BIC	aBIC	Entropy	LMRT (*p* value)	BLRT (*p* value)	Group size%
1-Class	2072.038	2089.197	2070.221	—	—	—	100
**2-Class**	**1801.325**	**1829.923**	**1798.296**	**0.993**	**0.0000**	**0.0000**	**55.1/44.9**
3-Class	1663.852	1703.889	1659.612	0.987	0.0581	0.0000	32.6/57.8/38/6
4-Class	1631.452	1682.928	1626.000	0.991	0.0848	0.0000	24.8/45.0/2.3/27.9

Bold indicates the selected model. AIC, Akaike Information Criterion; BIC, Bayesian Information Criterion; aBIC, sample-size adjusted BIC; LMRT, Lo–Mendell–Rubin adjusted likelihood ratio test; BLRT, Bootstrapped likelihood ratio test.

### 3.5 Differences in social disability across MCMQ profiles

Independent samples t-test was conducted to examine the differences in social disability scores between the two identified medical coping modes profiles. As shown in Table 4, a statistically significant difference was found between Adaptive Copers and Maladaptive Copers (*t* = 8.894, *p* < 0.001). Specifically, patients in the Maladaptive Copers group reported significantly lower levels of social disability (M = 0.24, SD = 0.844) compared to those in the Adaptive Copers group (M = 3.23, SD = 2.668).

**Table 4:** Differences in social disability scores between MCMQ-based latent profiles.

Subgroups	N	M ± SD	t	*p*
Adaptive copers	71	3.23 ± 2.668	8.894	<0.001
Maladaptive copers	58	0.24 ± 0.844		

### 3.6 Moderation analysis of perceived social support between MCMQ profiles and social disability

**Figure 2:**
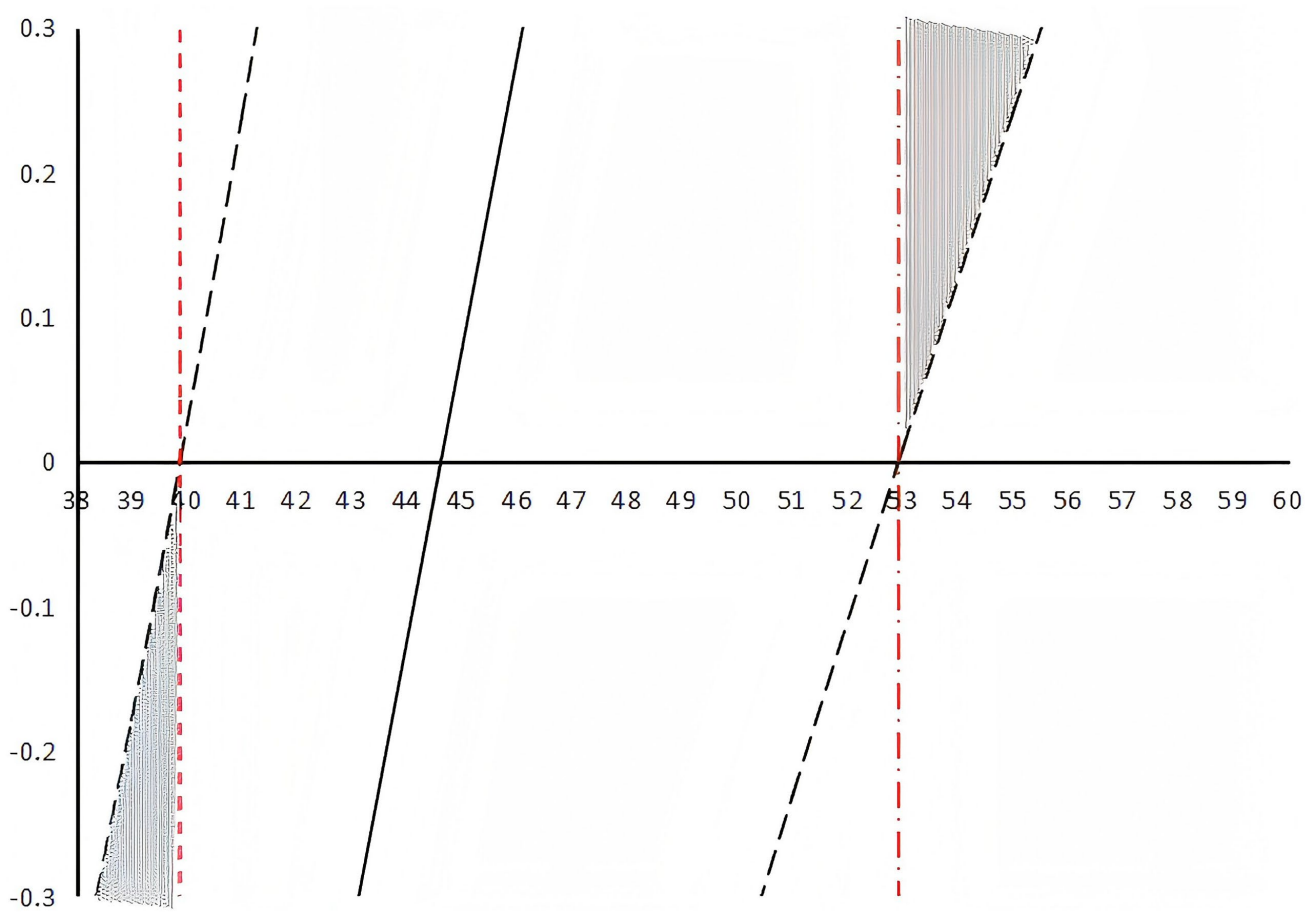
Johnson-Neyman plot of the moderating effect of social support.

The Johnson-Neyman technique was employed to precisely delineate the regions of significance for the moderating effect of perceived social support on the relationship between medical coping modes and social disability. As illustrated in Figure 2, perceived social support significantly moderated this relationship.

The analysis revealed two critical thresholds for perceived social support scores: 39.884 and 52.924. The moderating effect of social support was statistically significant (*p* < 0.05) when its score was below 39.884 or above 52.924.

## 4 Discussion

The present study utilized latent profile analysis and moderation analysis to investigate the heterogeneous patterns of medical coping modes among PCI patients and the moderating role of perceived social support in the relationship between coping modes and social disability. The main findings revealed two distinct coping profiles, significant differences in perceived social support between these profiles, and a significant moderating effect of perceived social support within specific thresholds. These results extend previous research by demonstrating the complex interplay between psychological coping mechanisms and social environmental factors in determining functional outcomes in PCI patients.

### 4.1 Interpretation of key findings

First, consistent with our hypothesis and previous research on coping heterogeneity in chronic illness populations (Condon et al., 2020; Guiry et al., 1987), medical coping modes were not uniform among PCI patients. LPA successfully identified two distinct subgroups: Adaptive Copers (characterized by higher confrontation and lower avoidance/resignation) and Maladaptive Copers (characterized by the opposite pattern). This finding aligns with studies suggesting that patients exhibit heterogeneous coping strategies following acute cardiac events (Alanazi et al., 2025; Yamaguchi et al., 2025). The nearly equal distribution between adaptive and maladaptive copers (55.1% vs. 44.9%) highlights a significant proportion of patients who may benefit from targeted psychological interventions to improve their coping strategies post-PCI.

Second, a significant difference in perceived social support scores was observed between the two coping profiles. Contrary to some prior research which typically links avoidance and resignation to poorer outcomes (Shimode et al., 2025; Liang et al., 2022), our study yielded a counterintuitive finding: the Maladaptive Copers group reported significantly lower levels of social disability. This phenomenon can be interpreted through the lens of the cognitive-transactional model of stress. In the short-term, post-discharge context of PCI recovery, avoiding illness-related stressors and resigning to the situation might serve as an immediate, emotion-focused regulatory strategy. By disengaging from socially demanding roles and activities, these patients may temporarily reduce performance pressure and perceived failure, thereby lowering their self-reported social disability scores (Abrams, 1984). However, this apparent ‘advantage’ is likely illusory and context-dependent. While avoidance may offer a protective buffer in the acute phase, substantial evidence indicates that it is associated with poorer long-term psychological adjustment, lower treatment adherence, and worse functional recovery in cardiac patients (Blikman et al., 2014). Thus, the lower social disability score in this group may not signify healthier adaptation but rather a maladaptive disengagement from social roles that is detrimental to holistic recovery over time.

Third, and most importantly, perceived social support played a significant moderating role in the relationship between medical coping modes and perceived social support, but its effect was not linear. The Johnson-Neyman analysis revealed that social support significantly moderated this relationship only at very low (<39.884) and very high (>52.924) levels. This U-shaped moderating effect represents a novel contribution to the literature. It suggests that for patients with moderate levels of perceived social support, their coping style may be the primary driver of social disability. However, for those with exceptionally high social support, the protective effect was so strong that it significantly weakened the negative impact of maladaptive coping on social functioning, consistent with the stress-buffering hypothesis of social support (Bekiros et al., 2022; Maffei et al., 2025). Conversely, for those with very low perceived social support, the sheer lack of external resources created a vulnerable context where the individual’s coping style became paramount. In this resource-scarce environment, relying on avoidance and resignation—strategies that inherently lack proactive problem-solving—exacerbated their risk of social disability, consistent with research on the compounding effects of low perceived social support (Kazukauskiene et al., 2021; Blikman et al., 2014). Notably, the non-significant moderating effect within the moderate range of perceived social support implies that for most patients, their intrinsic coping style may be the primary driver of social disability outcomes when environmental support is neither absent nor overwhelmingly present.

### 4.2 Implications for clinical practice

Our findings offer several implications for clinical nursing practice and patient support. Firstly, the identification of two distinct coping profiles suggests that healthcare providers, especially cardiac nurses, should routinely assess the coping strategies of PCI patients (Sumin and Shcheglova, 2023). Early identification of Maladaptive Copers is crucial, Clinicians should be aware that these patients may experience relatively mild disabilities after PCI, which may mask their need for psychological support. This approach is in line with the recent call for personalized medical care in cardiac rehabilitation (Claes et al., 2024). Building on this, we propose that a comprehensive assessment should not be limited to coping styles but also encompass the patient‘s perceived social support level. This dual-assessment approach forms the foundation for personalized intervention.

Secondly, and more specifically, interventions should be tailored based on the combined insights into the patient‘s coping profile and support system, moving beyond a one-size-fits-all model. For patients with very low perceived social support, interventions must be intensive and multifaceted, focusing not only on building adaptive coping skills but also on actively connecting them with community resources, peer support groups, or counseling services to enhance their perceived support (Czajkowski et al., 2022; Purcell et al., 2021). For patients with already high perceived social support, the clinical focus should shift toward optimizing support quality. This involves educating family members and friends on how to provide effective, autonomy-supportive encouragement rather than fostering dependency or overprotective behaviors that might inadvertently reinforce avoidance (Uchino, 2006). In essence, the moderating effect revealed in our study translates into a stratified clinical strategy: for the vulnerable group with scant resources, the priority is to build support; for the well-supported group, the goal is to optimize its quality to maximize its buffering potential against maladaptive coping.

### 4.3 Limitations and future directions

This study has several limitations. First, the cross-sectional design prohibits any causal inferences regarding the relationships between coping styles, social support, and psychological distress. Although significant correlations were found, the direction of influence remains undetermined. Future longitudinal research is needed to track the dynamic evolution throughout the recovery trajectory. Second, the generalizability of our findings is limited by the sampling strategy and our specific inclusion criteria. Although recruiting a homogeneous sample of young and middle-aged patients undergoing their first PCI from a single center enhanced the internal validity of our study, it remains unclear whether these results can be generalized to older patients, those with repeated PCI procedures, or those receiving care in other healthcare settings. Future multi-center studies with broader eligibility criteria are needed to validate our findings across the wider PCI population. Third, the generalizability of the findings is constrained by the sample characteristics. The relatively small sample size (*n* = 129) may limit statistical power, and the pronounced gender imbalance (over 90% male participants) means the results may not be representative of female populations. Finally, all measures relied on self-reported data. Incorporating objective indicators of functional outcomes and observational assessments of perceived social support in future studies would provide a more comprehensive data perspective.

## 5 Conclusion

In conclusion, this study makes two principal contributions to the understanding of psychological adaptation after PCI. First, by employing latent profile analysis, we established the heterogeneity in patients’ coping responses, identifying two distinct patterns: adaptive and maladaptive copers. This confirms that coping is not a uniform construct in this population. Second, we elucidated the complex, nonlinear moderating role of social support, demonstrating that its protective effect on the coping-disability relationship is most potent only at extreme levels. The counterintuitive finding of lower short-term disability in maladaptive copers further underscores the complexity of this interplay.

These findings highlight the imperative for healthcare professionals to move beyond a one-size-fits-all approach. Implementing routine assessments of coping styles and developing tailored psychosocial interventions—ranging from building coping skills in unsupported patients to optimizing support quality in those who are well-supported—are essential steps toward improving long-term functional recovery and overall well-being in this patient population.

## Data Availability

The original contributions presented in the study are included in the article/supplementary material, further inquiries can be directed to the corresponding author.

## References

[ref1] Abrams Herbertl. Stress, appraisal and coping. By Lazarus R. S. Folkman S.. (Pp. 445; illustrated; $31.95.) Springer Publishing: New York. (1984).

[ref2] Alanazi M. Deka P. Given C. Alanazi M. O. Given C. W. Lehto R. . (2025). Coping strategies and health-related quality of life in individuals with heart failure. J. Clin. Med. 14:3073. doi: 10.3390/jcm14093073, 40364104 PMC12072779

[ref3] Am R. Zuidersma M. de Jonge P. (2012). Myocardial infarction and generalised anxiety disorder: 10-year follow-up. Br. J. Psychiatry 200, 324–329. doi: 10.1192/bjp.bp.111.103549, 22403086

[ref4] Bauer D. Curran P. (2005). Probing interactions in fixed and multilevel regression: inferential and graphical techniques. Multivar. Behav. Res. 40, 373–400. doi: 10.1207/s15327906mbr4003_5, 26794689

[ref5] Bekiros S. Jahanshahi H. Munoz-Pacheco J. (2022). A new buffering theory of social support and psychological stress. PLoS One 17:e275364. doi: 10.1371/journal.pone.0275364, 36223401 PMC9555651

[ref6] Berg S. Rasmussen T. Thrysoee L. Berg S. K. Rasmussen T. B. Thorup C. B. . (2018). Mental health is a risk factor for poor outcomes in cardiac patients: findings from the national DenHeart survey. J. Psychosom. Res. 112, 66–72. doi: 10.1016/j.jpsychores.2018.07.002, 30097138

[ref7] Berkman N. Davis T. Mccormack L. (2010). Health literacy: what is it? J. Health Commun. 15, 9–19. doi: 10.1080/10810730.2010.499985 20845189

[ref8] Berlin K. Williams N. Parra G. (2014). An introduction to latent variable mixture modeling (part 1): overview and cross-sectional latent class and latent profile analyses. J. Pediatr. Psychol. 39, 174–187. doi: 10.1093/jpepsy/jst084 24277769

[ref9] Blikman M. Jacobsen H. Eide G. Blikman M. J. C. Jacobsen H. R. Eide G. E. . (2014). How important are social support, expectations and coping patterns during cardiac rehabilitation. Rehabil. Res. Pract. 2014:973549. doi: 10.1155/2014/973549, 25302122 PMC4180384

[ref10] Burg M. Abrams D. (2001). Depression in chronic medical illness: the case of coronary heart disease. J. Clin. Psychol. 57, 1323–1337. doi: 10.1002/jclp.1100 11590618

[ref11] Carver C. Scheier M. Weintraub J. (1989). Assessing coping strategies: a theoretically based approach. J. Pers. Soc. Psychol. 56, 267–283. 2926629 10.1037//0022-3514.56.2.267

[ref12] Claes J. Kuznetsova T. Cauwenberghs N. Cornelissen V. (2024). The changing landscape of cardiac rehabilitation and the power of personalized therapy. Front. Cardiovasc. Med. 11:1393217. doi: 10.3389/fcvm.2024.1393217, 38699581 PMC11063310

[ref13] Cohen S. Wills T. (1985). Stress, social support, and the buffering hypothesis. Psychol. Bull. 98, 310–357. 3901065

[ref14] Condon S. Roesch S. Clements P. Condon S. E. Roesch S. C. Clements P. J. . (2020). Coping profiles and health outcomes among individuals with systemic sclerosis: a latent profile analysis approach. J. Scleroderma Relat. Disord. 5, 231–236. doi: 10.1177/2397198320930138, 35382526 PMC8922625

[ref15] Czajkowski S. Arteaga S. Burg M. (2022). “Social support and cardiovascular disease” in Handbook of cardiovascular behavioral medicine. eds. Waldstein S. R. Kop W. J. Suarez E. C. . (New York, NY: Springer New York), 605–630.

[ref16] Feifel H. Strack S. Nagy V. (1987). Coping strategies and associated features of medically ill patients. Psychosom. Med. 49, 616–625. 3423168 10.1097/00006842-198711000-00007

[ref17] Frasure-Smith N. Lesperance F. (2008). Depression and anxiety as predictors of 2-year cardiac events in patients with stable coronary artery disease. Arch. Gen. Psychiatry 65, 62–71. doi: 10.1001/archgenpsychiatry.2007.4, 18180430

[ref18] Guiry E. Conroy R. Hickey N. Conroy R. M. Mulcahy R. (1987). Psychological response to an acute coronary event and its effect on subsequent rehabilitation and lifestyle change. Clin. Cardiol. 10, 256–260. 3581537 10.1002/clc.4960100409

[ref19] Hagger M. Koch S. Chatzisarantis N. Hagger M. S. Chatzisarantis N. L. D. Orbell S. (2017). The common sense model of self-regulation: meta-analysis and test of a process model. Psychol. Bull. 143, 1117–1154. doi: 10.1037/bul0000118, 28805401

[ref20] Hayes A. (2013). “Introduction to mediation, moderation, and conditional process analysis: a regression-based approach” in Introduction to mediation, moderation, and conditional process analysis: A regression-based approach. Ed. Hayes A. F. New York: The Guilford Press.

[ref21] Holt-Lunstad J. Smith T. Baker M. Smith T. B. Harris T. Stephenson D. (2015). Loneliness and social isolation as risk factors for mortality: a meta-analytic review. Perspect. Psychol. Sci. 10:227. doi: 10.1177/1745691614568352, 25910392

[ref22] Janca A. Kastrup M. Katschnig H. López-Ibor J. J. Jr. Mezzich J. E. Sartorius N. (1996). The World Health Organization short disability assessment schedule (WHO DAS-S): a tool for the assessment of difficulties in selected areas of functioning of patients with mental disorders. Soc. Psychiatry Psychiatr. Epidemiol. 31, 349–354. 8952375 10.1007/BF00783424

[ref23] Journault A. Plante I. Charbonneau S. Journault A.-A. Sauvageau C. Longpré C. . (2022). Using latent profile analysis to uncover the combined role of anxiety sensitivity and test anxiety in students' state anxiety. Front. Psychol. 13:1035494. doi: 10.3389/fpsyg.2022.1035494, 36619097 PMC9811949

[ref24] Kazukauskiene N. Bunevicius A. Gecaite-Stonciene J. Burkauskas J. (2021). Fatigue, social support, and depression in individuals with coronary artery disease. Front. Psychol. 12:732795. doi: 10.3389/fpsyg.2021.732795, 34744903 PMC8565598

[ref25] Kelter R. (2020). Bayesian alternatives to null hypothesis significance testing in biomedical research: a non-technical introduction to Bayesian inference with JASP. BMC Med. Res. Methodol. 20:142. doi: 10.1186/s12874-020-00980-6 32503439 PMC7275319

[ref26] Kristofferzon M. Lofmark R. Carlsson M. (2005). Coping, social support and quality of life over time after myocardial infarction. J. Adv. Nurs. 52, 113–124. doi: 10.1111/j.1365-2648.2005.03571.x 16164472

[ref27] Lawton J. Tamis-Holland J. Bangalore S. (2022). 2021 ACC/AHA/SCAI guideline for coronary artery revascularization: a report of the American College of Cardiology/American Heart Association joint committee on clinical practice guidelines. J. Am. Coll. Cardiol. 79, e21–e129. doi: 10.1016/j.jacc.2021.09.005 34895950

[ref28] Leventhal H Meyer D Nerenz D. The common sense model of illness danger. 1980. The common sense model of illness danger. Ed. S Rachman. Contributions to medical psychology (Vol. 2, pp. 7–30). Pergamon Press.

[ref29] Levine G. Cohen B. Commodore-Mensah Y. Levine G. N. Cohen B. E. Fleury J. . (2021). Psychological health, well-being, and the mind-heart-body connection: a scientific statement from the American Heart Association. Circulation 143, e763–e783. doi: 10.1161/CIR.0000000000000947 33486973

[ref30] Liang L. Liu Y. Shi Y. Liang L.-X. Shi Y.-J. Jiang T.-T. . (2022). Family care and subjective well-being of coronary heart disease patients after percutaneous coronary intervention: mediating effects of coping strategies. Int. J. Nurs. Sci. 9, 79–85. doi: 10.1016/j.ijnss.2021.09.006, 35079608 PMC8766782

[ref31] Maffei A. Scarpa E. Patron E. Sessa P. (2025). Social anxiety influences the stress-buffering potential of social presence: evidence from cardiovascular and affective reactivity under stress. PLoS One 20:e325303. doi: 10.1371/journal.pone.0325303, 40460062 PMC12132970

[ref32] Mavrides N. Nemeroff C. (2013). Treatment of depression in cardiovascular disease. Depress. Anxiety 30, 328–341. doi: 10.1002/da.22051, 23293051

[ref33] Phillips M. Zhang J. Shi Q. Phillips M. R. Song Z. Ding Z. . (2009). Prevalence, treatment, and associated disability of mental disorders in four provinces in China during 2001-05: an epidemiological survey. Lancet 373, 2041–2053. doi: 10.1016/S0140-6736(09)60660-7, 19524780

[ref34] Purcell C. Smillie S. Boon M. (2021). Effectiveness of social network interventions to support cardiac rehabilitation and secondary prevention in the management of people with heart disease. Cochrane Database Syst. Rev. 2021:CD013820. doi: 10.1002/14651858 36743076 PMC7611922

[ref35] Rao S. O Donoghue M. Ruel M. (2025). 2025 ACC/AHA/ACEP/NAEMSP/SCAI guideline for the management of patients with acute coronary syndromes: a report of the American College of Cardiology/American Heart Association joint committee on clinical practice guidelines. J. Am. Coll. Cardiol. 85, 2135–2237. doi: 10.1161/CIR.0000000000001397 40013746

[ref36] Richardson S. Shaffer J. Falzon L. Shaffer J. A. Krupka D. Davidson K. W. . (2012). Meta-analysis of perceived stress and its association with incident coronary heart disease. Am. J. Cardiol. 110, 1711–1716. doi: 10.1016/j.amjcard.2012.08.004, 22975465 PMC3511594

[ref37] Shen X. Jiang Q. (2000). Report on application of Chinese version of MCMQ in 701 patients. Chin. J. Behav. Med. Sci. 9:3.

[ref38] Shimode Y. Kitai T. Iwata K. Murai R. Miyakoshi C. Izawa S. . (2025). Impact of stress coping style on self-care behaviors and prognosis in patients with heart failure: a prospective longitudinal observational study. Int. J. Cardiol. 421:132865. doi: 10.1016/j.ijcard.2024.132865, 39622346

[ref39] Sumin A. Shcheglova A. (2023). Pathogenetic mechanisms underlying major adverse cardiac events in personality type D patients after percutaneous coronary intervention: the roles of cognitive appraisal and coping strategies. Diagnostics 13:3374. doi: 10.3390/diagnostics13213374, 37958270 PMC10648350

[ref40] Teng M. Wang J. Jin M. Yuan Z. He H. Wang S. . (2024). Psychological capital among clinical nurses: a latent profile analysis. Int. Nurs. Rev. 71:786–793. doi: 10.1111/inr.12918, 38051001

[ref41] Tiemensma J. Gaab E. Voorhaar M. (2016). Illness perceptions and coping determine quality of life in COPD patients. Int. J. Chron. Obstruct. Pulmon. Dis. 11, 2001–2007. doi: 10.2147/COPD.S109227 27601893 PMC5003510

[ref42] Uchino B. (2006). Social support and health: a review of physiological processes potentially underlying links to disease outcomes. J. Behav. Med. 29, 377–387. doi: 10.1007/s10865-006-9056-5, 16758315

[ref43] Wang J. Mann F. Lloyd-Evans B. Ma R. Johnson S. (2018). Associations between loneliness and perceived social support and outcomes of mental health problems: a systematic review. BMC Psychiatry 18:156. doi: 10.1186/s12888-018-1736-5, 29843662 PMC5975705

[ref44] Xiao S. (1994). The theoretical basis and research application of the social support rating scale. J. Clin. Psychiatry 4:3.

[ref45] Yamaguchi D. Asano Y. Kuwahara K. Izawa A. (2025). Coping strategies and changes in type D personality were associated with depressive tendency at 9 months after percutaneous coronary intervention. PLoS One 20:e316639. doi: 10.1371/journal.pone.0316639, 39804897 PMC11729923

[ref46] Yancy C. Jessup M. Bozkurt B. (2013). 2013 ACCF/AHA guideline for the management of heart failure. Circulation 128, 1810–1852. doi: 10.1161/CIR.0b013e31829e8807 23741057

[ref47] Yeh R. Secemsky E. Kereiakes D. Yeh R. W. Secemsky E. A. Kereiakes D. J. . (2016). Development and validation of a prediction rule for benefit and harm of dual antiplatelet therapy beyond 1 year after percutaneous coronary intervention. JAMA 315, 1735–1749. doi: 10.1001/jama.2016.3775, 27022822 PMC5408574

[ref48] Zhang M. (1998). Manual of psychiatric rating scales. Hunan: Hunan Science and Technology Publishing House.

[ref49] Zhang W. Nan N. Song X. Zhang W. Y. Song X. T. Tian J. F. . (2019). Impact of depression on clinical outcomes following percutaneous coronary intervention: a systematic review and meta-analysis. BMJ Open 9:e26445. doi: 10.1136/bmjopen-2018-026445, 31434764 PMC6707663

[ref50] Zhao Z. Mei Y. Wang X. Jiang H. Wang W. Lin B. . (2023). Meaning in life among nursing students: a latent profile analysis. BMC Nurs. 22:264. doi: 10.1186/s12912-023-01425-3, 37568145 PMC10416489

